# Supplementary material for: Genetic underpinnings of regional adiposity distribution in African Americans: Assessments from the Jackson Heart Study
Source: PLoS One. 2021 Aug 4;16(8):e0255609. doi: 10.1371/journal.pone.0255609 (PMC8336790; doi:10.1371/journal.pone.0255609)
Supplement: S2 Table — (DOCX) [file pone.0255609.s002.docx]

**S2 Table.** SNPs configurations used for calculation of polygenic risk scores under complementary approaches.

| Phenotype | All known SNPs* | All known SNPs (used under principal approach)** | Replicated SNPs (used under Approach 2)*** | Nominally significant (used under Approach 3) *** |
| --- | --- | --- | --- | --- |
| BMI | 3373 | 958 | 413 | 33 |
| WHR | 2051 | 464 | 166 | 12 |
| WC | 1475 | 502 | 81 | 8 |
| % Fat Mass | 106 | 57 | 48 | 4 |

*The list of available known SNPs for target traits at the time of study.

**A subset of known SNPs obtained after LD-pruning.

**numbers in the columns 3, 4 and 5 (from the left) represent subset of numbers from preceding columns. Abbreviations: **WHR**: Waist to Hip Ratio, **WC**: Waist Circumference, **BF%**: Body Fat Percentage, **SAT**: Subcutaneous Adipose Tissue
