# Supplementary material for: Genetic underpinnings of regional adiposity distribution in African Americans: Assessments from the Jackson Heart Study
Source: PLoS One. 2021 Aug 4;16(8):e0255609. doi: 10.1371/journal.pone.0255609 (PMC8336790; doi:10.1371/journal.pone.0255609)
Supplement: S3 Table — Estimates for all phenotypes other than percentage body fat (%BF). (DOCX) [file pone.0255609.s003.docx]

**S3 Table.** Polygenic Risk Score Validation (phenotypes other than percentage body fat (%BF)):

| Phenotype PRS vs. phenotype measures | Unit | Principle approach, β(p-value) * | Approach 2, β(P-value) | Approach 3, β(P-value) |
| --- | --- | --- | --- | --- |
| BMI | Weight(kg)/Height(m^2^) | **0.128(1.30×10^-72^)** | **0.151 (1.99×10^-40^)** | **0.381(2.46×10^-21^)** |
| WHR | Waist(cm)/Hip(cm) ratio | **0.002(1.60 ×10^-53^)** | **0.002(2.73×10^-19^)** | **0.005(4.32×10^-13^)** |
| WC | Waist circumference(cm) | **0.031(4.37×10^-37^)** | **0.248(3.18×10^-5^)** | **0.427(3.70×10^-2^)** |

*reported coefficients and p-values represent estimated change in measured phenotype per increase in number of risk alleles. Estimates are obtained linear regressions models. (Sample Size: N=2420). Abbreviations: **WHR**: Waist to Hip Ratio, **WC**: Waist Circumference, **BF%**: Body Fat Percentage, **SAT**: Subcutaneous Adipose Tissue, **β:** effect size (unit change per increase in number of risk alleles)
