# Supplementary material for: Genetic underpinnings of regional adiposity distribution in African Americans: Assessments from the Jackson Heart Study
Source: PLoS One. 2021 Aug 4;16(8):e0255609. doi: 10.1371/journal.pone.0255609 (PMC8336790; doi:10.1371/journal.pone.0255609)
Supplement: S4 Table — Spearman correlation coefficients were calculated. (DOCX) [file pone.0255609.s004.docx]

**S4 Table.** Matrix of correlation between observed phenotypic measures for Jackson Heart Study. Spearman correlation coefficients were calculated.

| **Spearman correlation coefficient, r (p-value)** | BMI | Waist Circumference | Waist to Hip Ratio | Body Fat % | Subcutaneous Fat Tissue | Visceral Fat Tissue | Visceral: Subcutaneous Fat Ratio |
| --- | --- | --- | --- | --- | --- | --- | --- |
| BMI | NA |  |  |  |  |  |  |
| Waist Circumference | 0.82  (2.20×10^-16^) | NA |  |  |  |  |  |
| Waist to Hip Ratio | 0.16  (3.33×10^-14^) | 0.57  (2.20×10^-16^) | NA |  |  |  |  |
| Body Fat % | 0.70  (2.20×10^-16^) | 0.49  (2.20×10^-16^) | -0.13  (1.72×10^-10^) | NA |  |  |  |
| Subcutaneous Fat Tissue | 0.83  (2.20×10^-16^) | 0.65  (2.20×10^-16^) | -0.04  (9.08×10^-2^) | 0.81  (2.20×10^-16^) | NA |  |  |
| Visceral Fat Tissue | 0.49  (2.20×10^-16^) | 0.63  (2.20×10^-16^) | 0.49  (2.20×10^-16^) | 0.29  (2.20×10^-16^) | 0.33  (2.20×10^-16^) | NA |  |
| Visceral: Subcutaneous Fat Ratio | -0.28  (2.20×10^-16^) | -0.02  (4.96×10^-1^) | 0.45  (2.20×10^-16^) | -0.45  (2.20×10^-16^) | -0.55  (2.20×10^-16^) | 0.54  (2.20×10^-16^) | NA |
