# Supplementary material for: Genetic underpinnings of regional adiposity distribution in African Americans: Assessments from the Jackson Heart Study
Source: PLoS One. 2021 Aug 4;16(8):e0255609. doi: 10.1371/journal.pone.0255609 (PMC8336790; doi:10.1371/journal.pone.0255609)
Supplement: S5 Table — They exhibit the proportion of adiposity traits’(BF%, SAT, VAT, VSR) variances were predicted by each individual PRS. (DOCX) [file pone.0255609.s005.docx]

**S5 Table.** Coefficients of determination (R^2^) for PRS, which exhibit the proportion of adiposity traits’(BF%, SAT, VAT, VSR) variances were predicted by each individual PRS.

| **Phenotype-PRS/Adiposity trait** | **BF%**  (%)* | **SAT**  (%) | **VAT**  (%) | **VAT: SAT R.**  (%) |
| --- | --- | --- | --- | --- |
| WHR | 0.9 | 1.0 | 2.0 | 0.1 |
| WC | 1.6 | 2.2 | 2.4 | 0.0 |
| BMI | 5.4 | 6.4 | 4.5 | 0.1 |
| BF% | 1.2 | 0.6 | 0.1 | 0.1 |

**WHR**: Waist to Hip Ratio, **WC**: Waist Circumference, **BF%**: Body Fat Percentage, **SAT**: Subcutaneous Adipose Tissue, **VAT**: Visceral Adipose Tissue, **VAT/SAT R**.: VAT to SAT Ratio.).

*The percentage of variation in the phenotype explained by the PRS.
