# Supplementary material for: Genetic underpinnings of regional adiposity distribution in African Americans: Assessments from the Jackson Heart Study
Source: PLoS One. 2021 Aug 4;16(8):e0255609. doi: 10.1371/journal.pone.0255609 (PMC8336790; doi:10.1371/journal.pone.0255609)
Supplement: S6 Table — Betas are reported for standardized inverse normalized values, followed by their respective p-values. Nominally statistically significant results (p<5.00×10^−2^) are in bold font. (DOCX) [file pone.0255609.s006.docx]

**S6 Table.** Associations between phenotype-PRS (columns), and measures of adiposity (rows). Betas are reported for standardized inverse normalized values, followed by their respective p-values. Nominally statistically significant results (p<**5.00×10^-2^**) are in bold font.

| **Phenotype-PRS/Adiposity trait** | **BF%**  β (p-value)  (95%CI) | **SAT**  β (p-value)  (95%CI) | **VAT**  β (p-value)  (95%CI) | **VAT: SAT R.**  β (p-value)  (95%CI) | **PRS calculation approach** | **Adjusted for BMI** |
| --- | --- | --- | --- | --- | --- | --- |
| WC | **0.6 (3.9×10^-2^)**  **(0.0, 1.2)** | 0.7 (9.6×10^-2^)  (-0.1, 1.5) | 0.8 (7.9×10^-2^)  (-0.1, 1.7) | 0.06 (8.7×10^-1^)  (-0.7, 0.8) | Approach 2 | No |
|  | 0.1 (9.0×10^-1^)  (-1.8, 2.0) | -1.1 (4.2×10^-1^)  (-3.7, 1.5) | 0.7 (6.2×10^-1^)  (-2.2, 3.7) | 1.7 (1.7×10^-1^)  (-0.7, 4.1) | Approach 3 |  |
| WHR | **0.5 (1.1×10^-2^)**  **(0.1, 1.0)** | 0.5 (7.7×10^-2^)  (-0.1, 1.1) | **1.0 (1.3×10^-3^)**  **(0.4, 1.6)** | 0.5 (7.2×10^-2^)  (-0.4, 1.0) | Approach 2 |  |
|  | **1.9 (1.6×10^-2^)**  **(0.4, 3.4)** | 0.7 (5.0×10^-1^)  (-1.4, 2.8) | 1.4 (2.3×10^-1^)  (-0.9, 3.7) | 0.7 (4.9×10^-1^)  (-1.2, 2.6) | Approach 3 |  |
| BMI | **1.4 (2.4×10^-29^)**  **(1.2, 1.7)** | **1.6 (4.0×10^-20^)**  **(1.3, 1.9)** | **1.3 (3.6×10^-11^)**  **(0.9, 1.7)** | -0.2 (1.3×10^-1^)  (-0.6, 0.1) | Approach 2 |  |
|  | **3.3 (2.5×10^-13^)**  **(2.4, 4.2)** | **4.1 (1.4×10^-11^)**  **(2.9, 5.2)** | **2.0 (3.8×10^-3^)**  **(0.6, 3.3)** | **-1.8 (1.1×10^-3^)**  **(-2.9, -0.7)** | Approach 3 |  |
| BF% | **2.5 (1.3×10^-9^)**  **(1.7, 3.3)** | **1.8 (1.0×10^-3^)**  **(0.7, 2.9)** | **1.0 (8.9×10^-2^)**  **(-0.1, 2.3)** | -0.7 (1.7×10^-1^)  (-1.7, 0.3) | Approach 2 |  |
|  | **4.2 (9.3×10^-4^)**  **(1.7, 6.7)** | 2.7 (1.2×10^-1^)  (-0.6, 6.0) | 0.9 (6.3×10^-1^)  (-2.8, 4.6) | -1.8 (2.5×10^-1^)  (-4.9, 1.3) | Approach 3 |  |
| WC | 0.3 (1.4×10^-1^)  (-0.1, 0.7) | 0.4 (9.3×10^-2^)  (-0.1, 0.9) | 0.6 (1.4×10^-1^)  (-0.2, 1.4) | 0.1 (7.7×10^-1^)  (-0.6, 0.9) | Approach 2 | Yes |
|  | 0.2 (7.6×10^-1^)  (-1.0, 1.4) | 0.2 (8.1×10^-1^)  (-1.3, 1.7) | 1.7 (1.7×10^-1^)  (-0.7, 4.1) | 1.5 (2.3×10^-1^)  (-0.9, 3.9) | Approach 3 |  |
| WHR | 0.2 (2.2×10^-1^)  (-0.1, 0.4) | -0.1 (7.2×10^-1^)  (-0.4, 0.3) | **0.6 (2.3×10^-2^)**  **(0.1, 1.1)** | **0.6 (2.4×10^-2^)**  **(0.1, 1.1)** | Approach 2 |  |
|  | **1.2 (1.6×10^-2^)**  **(0.2, 2.2)** | 0.2 (7.5×10^-1^)  (-1.0, 1.4) | 1.1 (2.6×10^-1^)  (-0.8, 3.0) | 0.9 (3.6×10^-1^)  (-1.0, 2.8) | Approach 3 |  |
| BF% | **1.4 (7.3×10^-8^)**  **(0.9, 2.0)** | **1.0 (1.2×10^-3^)**  **(0.4, 1.6)** | 0.3 (5.0×10^-1^)  (-0.7, 1.4) | -0.6 (2.2×10^-1^)  (-1.6, 0.4) | Approach 2 |  |
|  | **4.3 (1.2×10^-7^)**  **(2.7, 5.9)** | **1.9 (5.0×10^-2^)**  **(0.00, 3.8)** | 0.2 (8.8×10^-1^)  (-2.8, 3.3) | -1.8 (2.6×10^-1^)  (-4.8, 1.3) | Approach 3 |  |

**WHR**: Waist to Hip Ratio, **WC**: Waist Circumference, **BF%**: Body Fat Percentage, **SAT**: Subcutaneous Adipose Tissue, **VAT**: Visceral Adipose Tissue, **VAT/SAT R**.: VAT to SAT Ratio, **β:** effect size (% change in z-score per increase in number of risk alleles).

* Associations adjusted for age, sex and first 10 ancestry principal components.
