# Supplementary figures and images for: Genetic underpinnings of regional adiposity distribution in African Americans: Assessments from the Jackson Heart Study
Source: PLoS One. 2021 Aug 4;16(8):e0255609. doi: 10.1371/journal.pone.0255609 (PMC8336790; doi:10.1371/journal.pone.0255609)

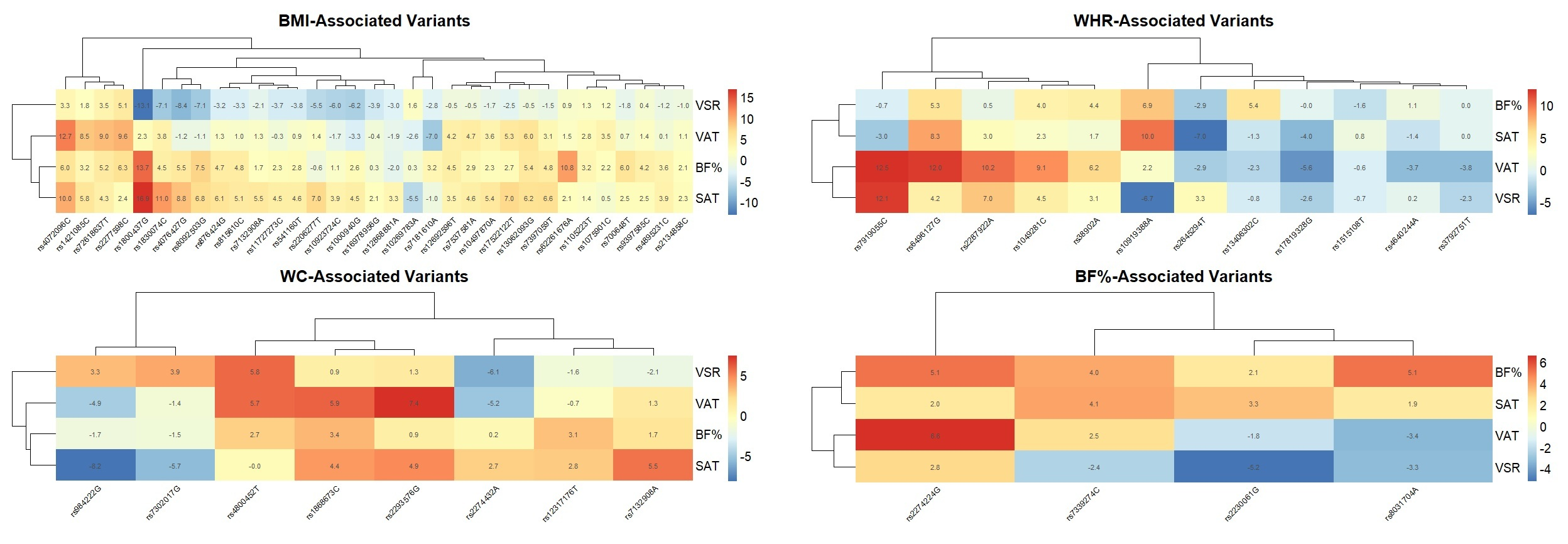

Supplement: S1 Fig — These SNPs were used for PRS calculation under approach 3 and represent polymorphisms with evidence of directionally consistent and statistically significant associations with their respective traits in JHS-genome wide assessment. Ward’s method used for SNP and trait clustering. (TIF) [file pone.0255609.s007.tif]
